# The experience of a nationwide Community of Practice to set up Regional Prevention Plans in Italy

**DOI:** 10.1186/s12961-017-0226-4

**Published:** 2017-07-27

**Authors:** Angela Giusti, Alberto Perra, Flavia Lombardo

**Affiliations:** National Center of Epidemiology, Surveillance and Health Promotion, National Institute of Health (Istituto Superiore di Sanità), Viale Regina Margherita 299, 00161 Rome, Italy

**Keywords:** Community of practice, Health policy planning, Planning process, Evaluation, Project cycle management, Prevention plan, Moodle, Knowledge, attitude and practice, SWOT analysis

## Abstract

**Background:**

In 2010, the Italian Ministry of Health decided to start the planning process to elaborate the National Plan of Prevention 2010–2012 jointly with the 21 Regions. The National Institute of Health was responsible for supporting regional planners (RPs) by an original participatory approach of a web-based Community of Practice (CoP) to set up their own Regional Plans of Prevention. In this paper, we summarise the theoretical framework adopted, the main phases characterising the lifecycle of the nationwide CoP, the evaluation approach adopted and its findings.

**Methods:**

Following the CoP theoretical framework from Wenger, an initial group of RPs were trained on Project Cycle Management as a planning method and thereafter they started interacting on a web-based Moodle platform for 8 months. The CoP evaluation mainly took into account aspects of ‘immediate value’, such as members interactions within the website, and several quantitative and qualitative tools were used to monitor changes over time. Data were retrieved from Moodle statistics or directly from the RPs by the means of a Knowledge, Attitude and Practice survey, a reaction survey, SWOT analysis and focus groups.

**Results:**

The level of individual RPs knowledge increased after the initial course from 55.7% to 75%, attitudes and competence perception about the planning process method also showed an overall favourable change. During the CoP life span, the number of members increased from the original 98 RPs to include up to 600 new members on the basis of spontaneous demand. From April 2010 to January 2011, the ‘vital signs’ of the CoP were monitored, including RP logins (13,450 total logins and 3744 unique logins), views (27,522) and posts (1606) distributed in 326 forum discussion threads. Data and information retrieved from quantitative and qualitative evaluation approaches proved to be useful for the management and follow-up of the CoP.

**Conclusions:**

The CoP experience was successful as 19 out of 20 Regions submitted their Regional Preventive Plan to their Ministry of Health within the due deadline. The CoP has proved to be an approach able to optimise resources and expertise, capitalising and generating new knowledge. However, more efforts should be deployed to define innovative ways to evaluate its values, tangible and intangible, as well as the return of investment.

## Background

In 2005, the Ministry of Health (MoH) elaborated and carried out the first National Prevention Strategy and Plan (2005–2007). More recently, following the main lines of the Action Plan for the Global Strategy for the Prevention and Control of Non-communicable Diseases (2008–2013) [1], as a first step, the MoH decided to commence the planning process to elaborate the National Plan of Prevention (NPP) 2010–2012 [2] in conjunction with the Sub-National Governments (21, thereafter called Regions). The NPP comprised strategies to improve prevention outcomes in the Italian population according to the four most important prevention areas, namely predictive medicine, primary prevention/health promotion, secondary prevention and disability prevention. Consequently, as a second step, the MoH entrusted the National Center of Epidemiology, Surveillance and Health Promotion (CNESPS), belonging to the National Institute of Health (*Istituto Superiore di Sanità*), to support Regional Planners (RPs) to set up their own Regional Plans of Prevention (RPP). Through an original participatory approach of a web-based Community of Practice (CoP), the RPPs were expected to be enforced by January 2011 conditionally to the ex-ante evaluation carried out by the MoH. Globally, NPP/RPPs, once approved, were to be funded for a total of 1440 million Euro to cover prevention projects for the period 2010–2012 and the money was allocated to the single Regions proportionally to their general population. The role proposed to the CNESPS was primarily justified by the need to promote, for the first time in Italy, a shared and standardised approach for the RPPs planning process (Project Cycle Management – PCM) [3] and a common understanding of the principles, strategies and outcomes of prevention. Based on its previous experience in promoting CoPs [4, 5], the CNESPS proposed a web-based National CoP involving all the planners and stakeholders identified by the Regions.

To achieve the utmost from this experience in order to improve its effectiveness in promoting and following other projects based on the CoP approach, the CNESPS identified two researchers (the first two authors of this article) to follow-up the new CoP and describe the main outputs and potential outcomes. The scientific literature was not particularly abundant in terms of specific tools for measuring CoP output and outcome nor for intangible outcomes such that researchers decided to utilise more common and familiar instruments like the Knowledge, Attitude and Practice (KAP) study tools to monitor the CoP and its development.

After an initial residential training set up by the CNESPS, the RPs made the commitment to set out some deliverables for each project included in their RPPs and, notably, the problem setting, the selection of the best evidence to support actions, the logical framework and the evaluation plan. Finally, planners were required to perform a cooperative, peer-reviewed evaluation, where a regional group of RPs was expected to make an ex-ante evaluation of another Region’s proposal.

In this paper, we summarise the theoretical framework, the main phases that characterised the lifecycle of the nationwide CoP aimed to set up the RPPs, the evaluation approach adopted and its findings with the objective of promoting further research on the CoPs knowledge and development in public health.

## Methods

### The theoretical framework of the CoP

Since the 1990s, Etienne Wenger has been describing a learning organisation, the CoP, based on the assumption that learning is an integral part of human nature, and that greater its effectiveness the more it is inserted in the context of participation in real life experiences rather than “*an individual process, with a beginning and an end, separated from the rest of our activities and the result of teaching*” [6]. According to the original definition, a CoP is “*a group of people who share a concern, a set of problems, or a passion about a topic, and who deepen their knowledge and expertise in this area by interacting on an ongoing basis*” [7]. In a CoP, members are voluntary adhering; membership can also be assigned and participation encouraged by management, but “*the kind of personal investment that makes for a vibrant community is not something that can be invented or forced*” [7]. During the last decade, the literature reported different experiences of CoPs also in the health sector, with some indications for measuring their results and for a better understanding of the potentialities to optimise the CoP’s outcomes [8, 9].

Traditionally, the CoP members are considered as sharing three fundamental elements, namely the Domain, which “*creates a common ground and a sense of common identity*”; the Community, which is strongly characterised by reciprocity and sense of trust; and the Practice, intended as the resources spontaneously made available by the CoP members but also the expected product, i.e. learning or other deliverables [10]. Many CoPs are spontaneous but they can be intentionally developed, as in our case, and formalised by organisations in order to steward and capitalise specific competences [10]. Other forms of working groups, such as project teams and networks, are mainly aimed at delivering products or services, accomplishing tasks or sharing information. The CoPs added value is the specific purpose “*to develop members capabilities and to build and exchange knowledge*” [11]. Based on the principles of social constructivism, it promises to be among the most efficient systems of co-construction, capitalisation and new knowledge generation.

### The nationwide CoP for RPPs planning process

In the context of our CoP experience, the shared Domain was the area of public health, health promotion, disease prevention and project planning. The Domain was also represented by the commitment that RPs, as CoP ‘core’ members, made to set up RPPs and the strong official recognition of their role in doing that. As health planners, they were familiar with the evidence-based approach for project planning. Moreover, as professionals, they improve their competencies in a continuous process based on reflexivity, valuing individual working experience [12, 13]. The Community was set up and formalised intentionally [7]. With the RPs being geographically dispersed around the country, the Community’s activity has mainly been based on the web, triggered by an initial residential training and followed by a final residential workshop. The Practice area was clearly defined by the CoP mandate, and included team working, context analysis, problem setting (mainly using epidemiological and qualitative data), goal and objectives identification, and the mapping of possible approaches.

Based on the CoP’s activity, RPs were expected to produce a shared draft of the RPP to be submitted to other regional colleagues and stakeholders to be finally approved by the Regions and the MoH.

This methodological CoP approach to support RPs was proposed by the CNESPS and approved by the MoH and by 20 out of 21 Italian Regions; thus, the CNESPS was appointed to perform the project.

### Phases of the learning experience and CoP’s life cycle

The CoP’s life cycle can be outlined in four phases. Table 1 shows the CoP’s timeframe, the learning settings, the main processes and lifecycle phases [7]. The first phase was constituted by a 5-day residential training for RPs to discuss and adopt the PCM approach, which has been promoted in recent years by the European Commission [14] as a common methodology for planning of the RPPs. A learner-centred approach was used to value participants’ experience, ideas and lessons learnt from each RP practice. Following the residential training, phase two consisted of starting up and implementing the national CoP. The CoP’s experience was based on a web2 environment, where RPs exchanged on methods and procedures, mainly on carrying out context analysis, making available the best evidence to support the intervention components of the projects, discussing the logical framework of different projects with similar objectives, conducting the analysis of risks associated with the implementation of projects and their sustainability, sharing good practices and lessons learned from previous preventive projects, and comparing and choosing the best approaches to project evaluation. Phase three was essentially devoted to the evaluation of CoP processes and outputs. Once the RPP proposals were submitted to the MoH, the RPs convened on closing the intensive phase of CoP exchanges. The web-based environment remained still accessible for out-of-project interactions for more than 3 years for residual spontaneous CoP communication (phase four) and for discussing the preparation of the following National Prevention Plan (2014–2018). The overall CoP learning experience has been accredited as post-graduate education by the University of Rome ‘Tor Vergata’ and considered equivalent to an annual Continuing Medical Education (CME) programme.

**Table 1:** Learning experience: timeframe, settings, Community of Practice (CoP) processes and lifecycle phases

Phase	Time	Learning setting	CoP main processes	CoP lifecycle phases
1	Apr–Jun 2010	5-day initial residential training (four editions)	- Reciprocal knowledge - Meanings and methods sharing - Identity building	CoP planning, coalescing and start-up
2	Sep 2010	On-the-job and constructivist web-based environment	- Fine-tuning of cooperative and collaborative procedures - Co-producing and exchanging parts of programmes	Active growth, sustain/renew
3	Jun 2011	1-day final residential workshop (two editions)	Evaluating common experience and outputs	Close
4	Post-project	Web-based environment still accessible	Out-of-project exchange ideas, materials, advice	Dispersed, memorable (residual spontaneous CoP)

A web environment based on Moodle 1.9 [15] was set up by the CNESPS to support the CoP activities. Based on the theory of social constructivism, Moodle is itself the expression of a worldwide CoP. The design of the web environment included synchronous and asynchronous communication tools (chat, instant messaging, forums), a database aimed to share resources (i.e. review of the evidence, grey literature, good practices), presentations, lectures and group works from residential workshops, survey modules to get feedback and to monitor ongoing activities and participant’s reactions, shared timetables, regional working areas for sharing and reviewing local outputs (i.e. drafts of PPRs), and online help system. Both individual RPs’ and CoP activities within the Moodle environment were monitored and evaluated by the CNESPS team.

### CoP members

According to Wenger’s description of the CoP participation levels [7], the initial CoP Core Group was constituted by the RPs, progressively complemented by active members like the community managers and the Project Technical Group. Some external experts also participated as occasional members while the MoH and the Regional Governments were peripheral members. All groups included intersectoral stakeholders. This natural frame of the CoP was reproduced in the web environment, by means of identifying specific areas reserved for each group or more general areas for comprehensive interactions. Particular attention was drawn to preserve the peer-to-peer approach, as it was considered that hierarchical relationships among members could jeopardise participation.

The website and the Moodle environment, notably the forum, were accessible to core members starting from May 2010. The end of October 2010 was the deadline given to RPs for posting an advanced draft of their RPP to carry out the ex-ante evaluation according to the PCM, while December 31 was the last date available for the Regions to submit their final RPP to MoH.

The ‘life’ of the CoP and the participation of the different type of CoP members were measured through the ‘vital signs’ (see below).

### Evaluation plan and tools

Following the framework from Wenger [16], mainly the aspects of ‘immediate value’ and, secondarily, of ‘potential value’ were considered, e.g. interactions that, within the website, RPs were developing during the project time. Alongside the formal CME training assessment approach, mainly based on the Kirkpatrick evaluation model [17], several quantitative and qualitative tools were used for CoP monitoring and evaluation. Data were retrieved from Moodle statistics or directly from the RPs by means of a KAP survey, a reaction survey, Strengths Weaknesses Opportunities Threats (SWOT) analysis and focus groups. Table 2 summarises the main evaluation objects, timing and tools for data collection.

**Table 2:** Main evaluation objects, data collection timing and tools

Phase	Evaluation object	Timing	Tools
1. Initial residential workshop	A. Baseline and evolution of KAP and perceived competence on PCM	Beginning and end of residential workshop	Structured KAP questionnaire
2. On-the-job and web-based environment	B. Evolution of Attitudes and Practices and perceived competence on PCM	Mid-term evaluation (after 4–6 months)	Structured KAP web-based questionnaire
	C. Contribution to CoP on the web: ‘vital signs’, individual contribution (quality and quantity)	During CoP life (after 6–8 months)	Data collection from Moodle platform
3. Final residential workshop	D. RPs reactions and opinions about the CoP experience and the regional process of planning	Beginning of final workshop During final workshop	Reaction survey (Happy Sheets) SWOT analysis in small groups and Focus Group

*CoP* Community of Practice, *KAP* Knowledge, Attitudes and Practices, *PCM* Project Cycle Management, *RPs* Regional Planners, *SWOT* strengths, weaknesses, opportunities, and threats

A KAP questionnaire was administered to the RPs at the beginning (T0) and at the end (T1) of the initial residential training and 4–6 months after the residential training completion (T2). The participatory evaluation of the planning/CoP experience was held in May to June 2011 during a final workshop.

The questionnaire main items were established to assess KAP and perceived competence in using the different PCM tools within the usual work setting of RPs. Knowledge was measured through 11 multiple-choice questions (only at T0 and T1), validated for difficulty, distractive and discriminatory indexes, of which six questions were on the use of the evidence for planning and five on PCM method. Attitudes and perceived competence were measured through a 5-level Likert scale. A frequency scale was used for the Practice of PCM tools. Analysis was carried out using Stata ver. 9 and changes in attitudes at the different times (T0, T1 and T2) were tested with the Wilcoxon signed-rank test. Due to the limited number of participants, determinants analysis, e.g. regional differences, was not performed.

The members’ contribution to the CoP activities (‘vital signs’) was measured through over time logins, accesses to forums (views and posts), and number of resources shared and exchanged.

At the end of the learning experience, the RPs reactions towards the CoP experience and the regional process of planning were described through ‘Happy Sheets’, SWOT analysis and Focus Groups. The Happy Sheets were analysed according to a 5-level Likert scale (from ‘completely happy’ to ‘completely unhappy’). Twelve SWOT analysis sessions were carried out involving all of the RPs. Two focus groups were conducted with a selected theoretical sample of RPs according to their level of participation to the CoP activity. The final evaluation through SWOT analysis and focus groups aimed to (1) gather the views of participants about the CoP; (2) describe strengths, weaknesses, opportunities and threats of the planning process; (3) identify the main reasons for participation or non-participation to the CoP; and (4) identify the CoP tangible and intangible output and outcomes.

All qualitative data were transcribed and analysed with the software NVivo 9.0 using a content analysis approach.

### Results

The CNESPS project lasted 2 and half years, from January 2010 to July 2012, but the period intensively involving the CoP was from April 2010 to May 2011. However, the official completion of the project was declared in 2014 and the data concerning the CoP experience was analysed thereafter. The main findings of the evaluation are presented according to the evaluation objects scheme proposed in Table 2.

The 20 participating Regions initially made available four to seven key professionals each, amounting to 98 RPs who attended the initial 5-day residential training, which was repeated four times to allow them all to participate. Approximately 40% of RPs reported having received a previous formal or CME course on planning methods and techniques, but only 15% specifically on the PCM method. On average, the level of individual knowledge increased from 55.7% to 75% (from T0 to T1), the use of evidence for planning increased from 59.1% to 71.7% (increase 12.6%), and use of the PCM method from 51.7% to 79.1% (increase 27.4%).

RPs attitudes at T2 compared with the initial level (T0) showed an overall favourable change towards PCM use (*P* < 0.001), which, when correctly applied, may improve project efficiency (*P* < 0.001) and effectiveness (*P* < 0.001). PCM is easy to use (*P =* 0.002) and potentially useful in order to involve stakeholders (*P =* 0.01). Nevertheless, no change was appreciable in the applicability of PCM at local level (*P =* 0.6), because PCM is considered of a certain complexity (*P =* 0.2), time consuming (*P =* 0.5) and requiring a qualified intersectoral, multi-professional and multidisciplinary team (*P =* 0.12).

The perception of becoming competent in using the PCM method shifted from T0 to T2 towards a better self-reliance for planning (*P* < 0.0001), carrying out a preliminary problem setting (*P* < 0.0001), setting up an objective tree (*P* < 0.0001), identifying an evidence-based strategy for the project (*P =* 0.019), assessing the quality of the evidence (*P* < 0.0095), setting up the logic model (*P* < 0.0001) and logical framework analysis (*P* < 0.0001), identifying project sustainability factors (*P =* 0.0001), conducting the project risk assessment (*P =* 0.0009), selecting and constructing appropriate project indicators (*P =* 0.11), and planning evaluation activities (*P =* 0.5).

During its lifespan, the number of CoP members increased from the original 98 RPs (core group) to include many new members on the basis of the spontaneous demand by other regional colleagues involved in the RPP planning process or on request by the Regional Governments. Furthermore, in many Regions, groups of planners working on specific prevention topics joined the web-based CoP to share methodologies and to involve local stakeholders in a broader participatory process of RPP planning. At the end of its active phase, the web-based national CoP embraced almost 600 members.

**Fig. 1:**
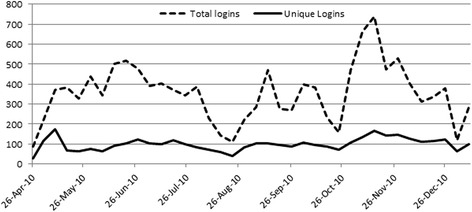
Regional Planners weekly accesses to the Moodle Platform, from end of April 2010 to January 2011

CoP ‘vital signs’ varied according to some events and deadlines. The website and the Moodle environment, notably the forum, were accessible to core members (RPs) starting from April 2010. As Fig. 1 shows, the initial raising trend in the logins (from May to end of June) is justified by the intense exchange of information between RPs in order to improve each other’s knowledge, to establish a good level of communication and to share resources. Most of the CoP members being on holiday explained the decrease in August, while the peak in early November is explained by the deadline given to RPs for posting a good draft of the RPP to be evaluated ex-ante internally to the CoP, while 31 December was the last date available for Regions to submit their own RPP to MoH. According to the project, after this last date, even if the RPs official commitment was to be considered as concluded, they decided to maintain a certain level of interaction within the forum, up to the end of March 2011. Over the period considered, the number of unique logins (i.e. weekly number of single users logged per day) and the total logins (i.e. weekly total number of logins) of CoP RPs, from the end of April 2010 to January 2011 (38 weeks), were 13,450 total logins and 3744 unique logins performed by the RPs, equivalent on average to 11 unique logins and 42 total logins per day, including Saturdays and Sundays.

**Fig. 2:**
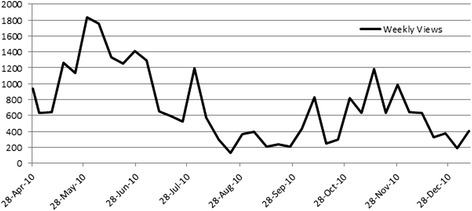
Core group members views in the RPs forum, from end of April 2010 to January 2011

**Fig. 3:**
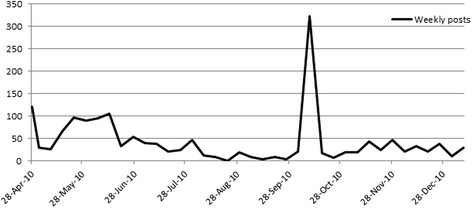
Core group members posts in the RPs forum, from end of April 2010 to January 2011

Altogether, 326 discussion threads were created in the dedicated forum by the RPs; Figs. 2 and 3 show their participation through the views (i.e. reading of other members’ posts without writing) and the posts (i.e. writing a post into the forum). In the same period, 27,522 views and 1606 posts were registered, corresponding, on average, to 103 views and six posts per day including Saturdays and Sundays, by the RPs. One or more files were attached in 8.7% (139/1606) of the total posts. Each participant’s activity was monitored and the decision was made within the core and active members to accept a certain degree of lurking, due to lack of confidence with the web environment or to other reasons that have been studied by the focus groups.

RPs were invited to post resources worth sharing due to being useful for planning health promotion and prevention activities, i.e. grey literature, reports, articles and website, in the CoP platform. After 8 months of CoP activity, 183 resources were uploaded by RPs in the web-based database.

RPs perception and opinions were explored during the final workshop. RPs feelings and opinions were studied with regards to three aspects through Happy Sheets, namely (1) the ongoing planning process within their own Regions; almost 75% perceived their responsibility and accountability in locally and methodologically conducting the preparation of the RPP even if more than 50% considered that the Region is not properly coordinating the overall RPP planning process. (2) The web-based environment (or Moodle platform) – by belonging to the CoP, 75% recognise to have had the opportunity to improve their knowledge; only one RP out of three considered themselves as a real CoP active member and 65% reckoned having received from the CoP more than they have offered; despite 50% feeling that the CoP environment inspired mutual trust, only 35% perceived a complete integration within the web-based CoP; exactly 50% of RPs perceived a climate of reciprocal trust within the CoP. (3) Opportunity to adhere in the future to similar initiatives – all RPs considered that such an experience (participating in a CoP) has to be reproduced in the future for other common (of the Regions) initiatives and 92% found the training experience conducted by the CNESPS useful. The main results of the SWOT analysis and the focus groups are summarised in Table 3.

**Table 3:** Main findings of SWOT analysis and Focus Group

	Strengths	Weaknesses
Internal	CoP - Constructivist environment - Participatory, non-judgmental, positive learning climate - Sharing of experiences within a multi-professional and multidisciplinary CoP - Intra- and inter-regional exchanges - Mutual knowledge and motivation - Development of sense of cooperation (joint efforts for shared goals) and collaboration (joint efforts for individual goals) - Construction of a sense of identity - Discussions focused on issues of interest ICT - Web platform as a working environment - ICT Help desk - Relevant to learning experience Outputs and outcomes of the CoP - Tangible (measurable) and intangible (not measurable) outcomes - Adaptive capacity in the face of complex tasks - Knowledge and practice of PCM - Identity, recognition (internal and external) - Common language, culture of planning design - Participation in local and platform	CoP - Poor representation of some relevant stakeholders - Opportunistic attitude, lurking - Some observers were not known by the CoP members - In some cases, hierarchical relationships inhibited plain participation - Low level of ‘active management’ of the group by CNESPS team - Interregional exchange not used at its full potential - The sense of not belonging for member joining the CoP later - Too many topics for discussion - Higher in the presence of CoP - Difficulties in valuing and giving external visibility to the intangible outcomes of the CoP ICT - Excess of messages from the web platform, poor capacity to manage them - Suboptimal use of the web platform with respect to potential - Obstacles in participation due to individual capacity (i.e. not confident with web environments)
	Opportunities	Threats
External	- Criteria for selection of participants (in some Regions) - Coordination by CNESPS team - Relevant to regional/national context of planning - Strong mandate - Possibility to develop the planning methodology at Regional level in wider groups, with local stakeholders - Relationships and networks created within the CoP have persisted after the experience	- Non-homogeneous criteria for the selection of CoP members (in some Regions) - Weak mandate at local level (in some cases) - Absence of regional managers and decision-makers - Central support weak (technical group) - Too much caution in sharing drafts of plans - Time constraints, no dedicated time for planning and peer-to-peer exchange - Poor external recognition of the value of the CoP as being in itself an outcome

*CNESPS* National Center of Epidemiology, Surveillance and Health Promotion, *CoP* Community of Practice, *ICT* Information and communications technology, *PCM* Project Cycle Management

## Discussion

The experience of setting up RPPs through a large-scale CoP constituted a remarkable challenge for the RPs and for the CNESPS. Some considerations concerning the evolution of the NPP in Prevention may justify this option.

In Italy, for the previous NPP (2005), the RPPs comprised a variable number of projects. In that first occasion, the Regions were not asked to use uniform planning methods or logical frameworks nor a common format for presenting the output of the project planning process. A recognised weak point of the old NPP (2005) was the limited efficiency of the planning process for which each Region employed its own experts. To give an example, a Region willing to set up a multi-strategy project aiming to prevent diabetes complications in the elderly, appointed a group of experts in order to find the best evidence (e.g. if guidelines were not available), the most cost-effective strategies, to define goals and adequate indicators, and to identify risks and assumptions. For some aspects, the planning process was independently repeated in all 21 Italian Regions, with a waste of energies that may have been better invested using collaborative and cooperative approaches. Apart from the pervasive ongoing spending review, the best use of the public resources is an ethical issue. On the other hand, the Italian regions affirm their autonomy in adapting and managing the strategies indicated at the national level (MoH). In a complex process such as a RPP, these issues need to be properly considered and addressed.

Conversely, for the NPP 2010–2012, the continuous and intensive exchange between RPs enabled them not only to set up RPPs according to a standardised methodologic approach but also to propose, for example, original project performance indicators that were consequently adopted by several regional groups or by the entire CoP. Many other examples of knowledge generation were offered by the CoP. In the case of cancer screening, some regions were able to share useful methodological approaches, helping other regions with lower levels of performance to understand and improve their projects with promising, more cost effective activities.

In our experience, a large-scale CoP for planning sub-national prevention plans was effective in capitalising previous regional experiences, sharing understanding, planning and evaluation approaches based on the PCM.

The findings of the evaluation contribute to explaining the high voluntary participation level in the CoP activities by the RPs and the feeling of efficiency in the planning process that led to most of the Regions setting up their RPP in only 6–8 months, even surprising the same RPs. They are also aware of acknowledging the return of investment, as stated by a participant: “*the time I spend to share the experience of my Region in cardiovascular diseases prevention, our set of indicators and our lessons learned is then refunded when I need to set actions, for example, to improve access to cervical cancer screening, if other members of the CoP have provided their own experience and resources.*”

Spending review, ethics, equity, sub-national and local autonomies are key points not only in Italy but also across different EU Countries. When translating a policy into national or subnational programmes, the planning process is a crucial starting point, where active participation of all stakeholders should be promoted from the very beginning and kept active throughout the process [18].

Despite the initial feeling of work overload brought about by the PCM method prescribing an initial and intense stakeholders participation in the planning process, RPs tuned in with this principle even if a certain variability was observed between different Regions.

A study [19] that performed a critical appraisal of the RPPs 2010–2012, found that geographical differences in the quality of the RPPs and the opportunity of assessing the planning process may strengthen public health capacity for prevention. According to our experience, the CoP was felt by RPs as an opportunity for the Regions with less capacity in prevention planning processes to gain experience from others more skilled, even if gaps still exist. In the future, to set up the next RPPs, it will be advisable to have a national follow-up of some critical steps of the regional planning process to avoid, for example, a lack of consideration of the principles of evidence-based prevention and to effectively tackle health inequalities [19, 20], as well as to retrieve the utmost from the national health behavioural surveillance systems well established in Italy [21] in order to monitor inequalities and outcome progress in prevention.

As a whole, the formation of a CoP to set up RPPs was very well accepted and supported by RPs. The usefulness of the web-based platform was confirmed by the increasing number of enrolled members (from 98 to over 600, after 8 months) that also obliged the CoP members and managers (CNESPS) to define more precise rules for accepting new participants.

The CoPs are mainly spontaneous and the ‘intentional’ approach of creating a CoP, as in our case, is not in itself a guarantee of success. In our experience, however, the RPs participated enthusiastically when they had received a strong mandate from their regional administration and were allowed a protected time to devote to the CoP activities.

The management of the CoP is not the object of this article, but aspects concerning a certain high rate of lurking and a weak coaching support were indicated by RPs during the focus groups.

Scarce information is available in the literature to compare participation level on the web-based platform by RPs. Most RPs showed a good level of familiarity with the Moodle platform, documented by a high number of logins during the functioning period of the platform as well as by a very intensive participation (either reading or writing) in the discussion threads, although, as expected, with a high variability between different RPs.

Due to the poor available evidence, the CoP performance and outcome evaluation was a useful learning experience for CNESPS.

Once the shared features of a CoP are clear (Domain, Community and Practice), it is essential to identify the outcome, especially for the Practice area, in order to perform an evaluation, and decide on the specific evaluation objects. Possibly, these are to be included within the ‘tangible’ CoP results and consequently a quantitative evaluation approach is to be identified. In our case, the KAP proved to be acceptable by RPs and easy to use as it was administered directly from the web-based Moodle platform. If the questionnaire is well framed and previously tested (sometimes many items are available from literature) it renders data easy understandable and allowing carrying out monitoring of Cop members’ changes over time.

In our experience, the use of a Likert scale to measure attitudes was useful to allow researchers to have a quantitative dimension to be statistically tested, but absolutely not sufficient to assign the right value and weight to CoP members opinions, point of views and cultural traits to explain the particular evolution of the CoP and the achievement of its outcomes, especially those called ‘intangible’. Most of the qualitative approaches to the evaluation (Focus groups, SWOTs and happy sheets) were utilised at the end of the experience but, due to the importance of the expected outcome (setting up RPPs) and the personal investment of the CoP members, possibly a mid-term qualitative approach would have allowed a better CoP management.

The systematic use of Moodle platform statistics allowed monitoring of CoP ‘vital signs’ and triggering platform managers’ interventions in case of inadequate use of the platform instruments or to strengthen CoP members interest in case of low participation to a particular important discussion thread or to encourage people to share resources or ideas.

Based on this nationwide experience, the National Institute of Health has progressively promoted the CoP approach for many projects as an effective strategy to enhance a continuous exchange of experiences, lessons learned and good practices in the field of communicable and non-communicable diseases. These projects include, at the regional level, in applied epidemiology, prevention and health promotion training, breastfeeding programmes and pharmacovigilance [22]; at the national level, in the field of elderly population surveillance and for preparing the National and Regional Plans 2014-2018 [22]; and at the European level, for the JA-CHRODIS - Work Package 7, gathering 28 partners from different countries [23, 24], and ASSET, joining 14 partners from 11 European countries [25].

## Conclusions

From a formal point of view, the support given by the CNESPS to the Regions to set up their RPPs through a nationwide CoP of RPs was successful as 19 out of 20 Regions submitted their RPP to the MoH within the due deadline.

It has to be considered that partaking in a CoP is time-consuming and regional decision-makers and health professionals need to be convinced that it is an investment, both for individual professional development and for the performance of the services they belong to. The practice of the CoP members, the deep knowledge of their different contexts, their experience in terms of transferability of good practices, the lessons learned from the practice, the sense of trust and mutual recognition of competence are at the same time intangible values and outcomes, as well as the RPs perception that the planning process is crucial for the effectiveness of the prevention.

The CoP has proved to be an approach able to optimise resources and expertise, capitalising and generating knowledge. This capital, in its tangible and intangible forms, must then be made available for similar experiences and further ‘cultivated’. CoPs are effective to exchange and generate high value knowledge and are widely used by national and international organisations [26–28]. More efforts should be deployed to define and study innovative ways to evaluate its values as well as the return of investment.

## Abbreviations

CNESPS: National Center of Epidemiology, Surveillance and Health Promotion
CoP: Community of Practice
CME: continuing medical education
KAP: knowledge, attitude and practice
MoH: Ministry of Health
NPP: National Plan of Prevention
PCM: Project Cycle Management
RPP: Regional Plans of Prevention
RPs: Regional Planners
SWOT: strengths, weaknesses, opportunities and threats
